# Exploring caregiver experiences of raising a child with feeding difficulties: a qualitative study

**DOI:** 10.3389/frcha.2026.1753720

**Published:** 2026-04-29

**Authors:** Veena Satheesh, Bradley Scott Bloomfield, Rachel June Bartlett

**Affiliations:** Educational Psychology and Counselling, Monash University, Melbourne, VIC, Australia

**Keywords:** Australia, caregiver perspectives, feeding difficulties, framework analysis, interviews

## Abstract

**Introduction:**

Feeding difficulties can significantly disrupt family routines and contribute to caregiver stress, yet limited research has explored the lived experiences of Australian caregivers navigating this challenge.

**Methods:**

The objectives of this exploratory qualitative study was to investigate the experiences of six Australian caregivers raising children aged 2-18 with feeding difficulties. Six semi-structured interviews were conducted and analysed using framework analysis.

**Results:**

Four themes emerged: (1) caregiver concern for child wellbeing, including nutrition, emotional health, and social functioning; (2) impacts on the family, including disrupted mealtimes, strained relationships, and intergenerational tension; (3) challenges and strategies used to manage feeding difficulties; and (4) needs for support, highlighting service gaps, barriers to care, and mixed views on telehealth. Findings suggest the emotional and logistical burden of feeding difficulties on families and the importance of responsive, family-centred approaches.

**Discussion:**

These findings provide preliminary qualitative insight to inform future research and service development. Health professionals should consider both the psychosocial context and practical needs of caregivers when supporting feeding concerns.

## Exploring caregiver experiences of raising a child with feeding difficulties: a qualitative study

Feeding is an integral part of parenting, with early feeding practices setting a foundation for future child development (1). Difficulties with feeding can be seen as existing on a spectrum, ranging from mild feeding difficulties to feeding disorders that are clinically significant (2). Feeding difficulties are commonly characterised by restricted preferences, difficulties with textural, smell and taste properties, and limited intake of food; however, there is great diversity in presentation (3). Without adequate identification and treatment, prolonged feeding difficulties can impact the child's growth, skill development, and increase the likelihood of medical complications and illness (4). Feeding difficulties of varying severities have also been shown to have negative impacts on family life with caregivers often experiencing secondary impacts of their child's feeding difficulties (5, 6). This includes strains in the caregiver-child relationship, difficulties within their social lives, and being at a heightened risk for developing mental health problems (7, 8). Despite growing recognition of these challenges, limited research has explored caregivers' lived experiences around raising children with feeding difficulties.

Caregiver feeding practices should be considered relationally alongside the child's behaviour to address feeding difficulties, as feeding involves a bidirectional dynamic between caregiver and child (9, 28). Previous qualitative studies have provided significant insight into areas of caregiver concerns, types of food fussiness, impacts on the family and the emotional strain related to raising a child with feeding difficulties (8, 10, 11). Caregiver help-seeking behaviour, however, highlights a gap in knowledge around approaches to feeding. Caregivers seeking support online have sought out practical strategies to resolve feeding issues (12). Alternatively, caregivers calling a crisis help-line were seeking support around practical and quick solutions to the feeding issues they were experiencing (5).

In a study investigating stress amongst caregivers of children with paediatric feeding disorders, the authors report high levels of defensive responders among participants [Abidin, (29), as cited in Silverman et al. (13)]. The authors purport that this form of reporting bias led to the under-reporting of stress, particularly in caregivers of children with internalising behaviour problems. The present study therefore uses qualitative semi-structured interviews to explore caregiver experiences of feeding difficulties, building rapport with participants prior to asking questions about potentially difficult challenges for caregivers (14).

Given the critical influence of culture on feeding practices (1), caregiver experiences of feeding may vary across cultural contexts. Limited research has been done to examine the perspective of Australian families, aside from a recent study by Chilman et al. (10). Chilman et al. (10) interviewed 10 Australian caregivers of young children who are *extremely fussy eaters* aged between 2 and 6. This allowed for a focused investigation on the impacts of extreme fussiness in young children, whilst still demonstrating variability amongst caregiver participants. The participants vacillated between stress and optimism for the future for their children. While this allowed for a focused investigation of the impacts of extreme fussiness in young children, the present study had a less restrictive inclusion criteria capturing the heterogeneity of feeding difficulties across children, as recommended by Chilman et al. (10). This approach was exploratory and was not intended to compare developmental stages.

### Aims

Despite the high prevalence of feeding difficulties in children, few studies have explored the lived experiences of caregivers raising children with feeding difficulties in Australia. The aim of this exploratory qualitative study was to examine how Australian caregivers describe the impact of feeding difficulties on family life and to identify perceived needs for support. This study addressed two research questions: (1) How do caregivers perceive the impacts of feeding difficulties on the child and family? and (2) How can support services be shaped to meet the needs, values and expectations of caregivers? Findings are intended to provide preliminary qualitative insight to inform future research and professional practice for children with feeding difficulties.

## Method

### Research design

A qualitative research design was employed using framework analysis (15) to examine data collected through semi-structured interviews. Framework analysis was selected due to its structured yet flexible approach, allowing systematic comparison across participants while accommodating both *a priori* research questions and inductively derived insights (16, 17). This approach is well suited to applied health research and facilitates transparent coding through the development of an analytical framework and matrix-based comparison.

### Ethical considerations

This study was approved by the [blinded for review] Human Research Ethics Committee. Interviews were conducted online via Zoom between July and August 2024. Audio recordings were securely stored, and transcripts were generated using Zoom's automated transcription service and manually cross-checked against recordings to ensure accuracy. Interviews ranged from approximately 20–60 min. Shorter interviews were information-rich and focused on the interview domains; no repeat interviews were conducted. Semi-structured interviews included open-ended questions and follow-up probes tailored to participant responses. Upon completion, participants received a $100 AUD gift voucher as compensation for their time. The value of the voucher was determined in line with university guidelines to acknowledge participant contribution without constituting undue inducement.

Each participant was sent an explanatory statement regarding the nature of the interview and provided informed consent via electronic signature. They were also provided with contact details for support services if they experienced discomfort during participation. Participants provided their contact information so a member of the research team could schedule an interview.

### Positionality

The first author was a student undertaking a research thesis for a graduate diploma of professional psychology under the supervision of the second author. Two of the researchers (authors 2 and 3) have backgrounds in the field of feeding difficulties and behaviour analysis. Reflexive discussions were undertaken to consider how researchers' professional backgrounds in feeding difficulties and behaviour analysis may shape interpretation. All researchers kept a journal throughout the interview process and data analysis stage, which was consulted after familiarisation, coding and during theme development. As the researchers come from various cultural backgrounds, there was discussion around personal cultural experiences with food and how this influenced interpretation of mealtime traditions reported by participants.

### Sampling, recruitment, and participants

Convenience sampling was employed. Participants were recruited through two pathways: (1) invitation to survey respondents from a larger study who indicated interest in follow-up interviews, and (2) distribution of a recruitment poster via Australian allied health and behaviour support services and social media platforms.

Participants were required to be living in Australia and have a child between the ages of two and 18 with feeding difficulties or food selectivity; a diagnosis for a feeding disorder was not required. Eleven caregivers initially expressed interest. Six were unable to be contacted after multiple attempts via phone and email and were therefore excluded. One additional participant came from a related project and was approved for use in this study. A total of six caregivers participated in this study, reporting on 8 children.

Background information was extracted from the transcripts based on what caregivers disclosed during the interview (see Table 1). Demographic variables were not directly collected otherwise to protect participant anonymity, given the small sample and potentially identifiable nature of feeding presentations. Two caregivers (Cath and Carly) reported on more than one child with feeding difficulties; however, the unit of analysis was the caregiver.

**Table 1:** Caregiver demographic details.

Caregiver	Relationship to child	Child	Siblings	Onset of feeding concerns	Child's age	Child's gender	Child's reported diagnoses
Cath	Mother	1	Yes (2)	Early childhood	9	M	Autism, ADHD, Anxiety
		2	Yes (2)	Early childhood	5	M	Autism
Holly	Mother	1	Yes (2)	2	N/R	F	Autism, anxiety
Alex	Father	1	N/R	N/R	N/R	F	N/R
Eric	Father	1	N/R	9	17	M	N/R
Carly	Mother	1	Yes (1)	2	9	F	N/R
		2	Yes (1)	N/R	5	M	N/R
Maddie	Mother	1	Yes (1)	2	7	F	Anxiety

Caregiver participant names have been revised as pseudonyms. “Early childhood” refers to feeding difficulties reported to have early onset but no specific age. N/R, not reported.

### Data analysis

#### Familiarisation

All researchers engaged in familiarisation through conducting interviews, reviewing audio recordings, cleaning transcripts, and repeated reading. Initial analytic notes were recorded, including reflections on emerging patterns and researcher reactions. Transcripts downloaded from Zoom were manually cross-checked with the audio recordings and corrected if any errors were present, to ensure they were verbatim. Each researcher also kept a record of initial impressions of the data; this included notes on personal reactions to the data and patterns in responses across the interview. These were reviewed in conjunction with contextual notes that were made during the interviews.

#### Coding

Two researchers independently coded the same initial transcript to develop preliminary codes. A code is a label given to a verbatim extract—or “reference” of the data. Codes were refined through discussion and clustered into categories to form the working analytical framework. Coding incorporated both deductive elements informed by the research questions and inductive insights emerging from participant accounts. The agreed framework was then systematically applied to subsequent transcripts using NVivo 14. Where new concepts arose, the framework was updated, and earlier transcripts were revisited to ensure consistency.

A framework matrix was developed to summarise coded data by category and participant, facilitating comparison both within and across cases (17). This process was supported using NVivo's matrix function and Excel for organisation.

#### Mapping and interpretation

After developing the framework matrix, the researchers met to discuss initial interpretations, the relationship between the data and proposed themes. Themes were developed through collaborative interpretation of the framework matrix. In this process, the researchers reviewed patterns, refined thematic groupings, and discussed the coherence of the dataset in relation to the research questions.

#### Trustworthiness

Researchers employed several strategies to enhance analytic rigor with a focus on trustworthiness. Independent coding of an initial transcript supported consistency in framework development. Iterative refinement of codes and themes occurred through regular research team meetings where the research team debriefed identified codes and themes. Reflexive journaling was maintained throughout data collection and analysis to acknowledge potential researcher influence. The structured framework matrix provided an audit trail, enabling transparent tracking of coding decisions and theme development.

## Results

Framework analysis identified four themes and associated subthemes (Table 2). Themes reflect how caregivers understood feeding difficulties as influencing child wellbeing, family functioning, day-to-day management, and support needs.

**Table 2:** Summary of themes and subthemes.

Theme	Subtheme	Description	P (*n*)
Caregiver concern and child wellbeing	Child wellbeing	Caregiver concern for impacts on the child's mental, physical and social well-being	6
	Future independence and participation	Caregiver concern for child's independent functioning in future or social situations	4
Impacts on the family	Family difficulties	Impacts of feeding difficulty on immediate family members, and/or on relationships with extended family	6
	Strains on the caregiver-child relationship	Caregiver distress arising from management of feeding difficulties	6
	Intergenerational expectations	Familial traditions, beliefs and expectations around food and mealtimes	4
Challenges and strategies	Feeding-related challenges	Specific child behaviours, preferences, and characteristic that make feeding challenging	6
	Social situations	Arrangements to address feeding difficulty in social situations	5
	Efforts to encourage eating	Specific tactics used others to encourage the child to eat	6
Needs for support	Barriers	Factors that limited access to or reduced willingness to obtain support for the child's feeding difficulties	4
	Needs	Specific help desired from support services	6
	Telehealth	Previous experiences and potential for future use of online support services	6

“P”, number of participants that discussed content described in subtheme (*n* = 6).

### Caregiver concern and child wellbeing

This theme captures caregivers' understanding of feeding difficulties as not only an impact on physical health, but also to wellbeing, social participation, and future independence for their children. Caregivers often described feeding concerns beyond food itself, and into wider functioning and identity. Diet was the most prevalent concern; caregivers worried that the limited variety and consumption of foods was affecting their nutritional intake. Some caregivers thought that nutrition had flow-on effects on other aspects, such as behaviour or engagement; Carly described this by saying, “You haven't had enough food intake to sustain you for the school day”. Caregivers also had concerns about how their child was emotionally affected by the difficulties they experienced. A few caregivers noted that personal characteristics of their child impacted feeding and other aspects of their lives too; this included a lack of independence—requiring prompts, and anxiety—affecting the ability to eat with others. Interestingly, caregivers noted an awareness in their children of their own struggles with food. Cath stated, “He’ll frame it as…everyone struggles with something, I struggle with eating.” For some families, feeding difficulties had become a part of the child's self-narrative shaping how they interpreted their own differences. There was also discussion around children's perceptions of interventions; for example, Maddie discussed extensive involvement in therapy being tiresome for her child: “She’ll even eyeball me sometimes like ‘really, doing this again?’” Caregivers interpret this therapy fatigue as both a burden of the complex service involvement and the unintended costs of therapy on children.

Caregivers also worried about how their child would cope independently in situations, and notably, expressed the value of self-advocacy. “I just really want and need her as she gets older, to be able to communicate when she's unable to eat” (Holly). For some, there was concern about how their child's eating habits and abilities would affect them in future situations. For example, Maddie talked about how schooling would not continue to accommodate the length of time she needed to complete a meal: “All the kids would sit down and eat. Then they’d finish that, go outside and play but [child] is still at the table eating.” Eric shared feelings of worry about how his child is perceived by friends and how accepting prospective partners may be to his difficulties. For caregivers who were not seeing improvements in their child's eating habits, there was concern that difficulties would not be resolved. One caregiver, Maddie, reflected on this persistence to the feeding difficulties; “They’re not going away. And they are progressively, I suppose, enhancing”. Persistent or worsening difficulties were described as generating uncertainty about long-term prognosis and reinforcing anticipatory anxiety about adulthood.

### Impacts on the family

This theme reflects the relational and systemic impacts of feeding difficulties on the family system, including caregiver distress, disrupted routines, competing demands across siblings, and negotiation of mealtime expectations within family networks. Every caregiver reported experiencing some level of distress because of their child's feeding difficulties. Most discussed feeling stress or anxiety around mealtimes, and many felt that these negative feelings strained their relationship with their children. Maddie explained, “It causes us a lot of angst. And I can see that it's also translating on (child) and (sibling).” Caregivers described stress as spreading across the household, affecting sibling dynamics and caregivers’ perceived capacity to support their children during meals. Certain issues—such as food waste, particularly upset a few caregivers; Alex said, “It's more like I’m just wasting my money.” For one Maddie, extensive time with one child meant she could spend less on another: “It's exhausting for her sister who will finish her meal… [and] want to play or want my engagement.” Caregivers of multiple neurodiverse children reported high levels of stress, with one caregiver, Holly, experiencing caregiver burnout: “The information related to each child is overwhelming.” However, for Eric, a positive implication that arose from the extended time spent on addressing his child's feeding difficulties was that he felt closer to his child: “There's this bond that comes from parent and child being together.”

Caregivers also described intergenerational differences in mealtime expectations, often positioning their own approach as more flexible and responsive than the “finish your plate” norms they experienced growing up. Caregivers showed flexibility by offering a variety of foods to their children and discussed having less stringent mealtime expectations for their children than their caregivers had for them. Caregivers also voiced the importance of respecting their child's way of communicating satiety: Carly described, “A culture that we’ve really tried to avoid is the ‘finish your plate’.” Often, they addressed this by educating grandparents about their boundaries and expectations of their children, but combative beliefs sometimes caused tension when eating with the extended family—Cath noted, “Their grandmother will get upset if they don't eat”, whereas Holly responded, “It's a fine line of educating and not offending”. The inclusion of extended family members often intensified the complexity of managing feeding difficulties, as support was sometimes accompanied by conflicting expectations.

For many children, factors in their home environment affected their eating behaviour (see Table 3). Caregivers often addressed this by preparing separate meals for their children or eating at different times, which they felt was not ideal: according to Cath, “It's very resource intensive, and sometimes the three of them won't even eat the same thing”. Maddie articulated the desire to preserve her previous traditional family mealtimes, in which the whole family ate the same food together; “It's strange we don't do that, and I kind of wish we did.”

**Table 3:** Overview of feeding difficulties reported by caregivers.

Feeding difficulty	Verbatim example
Won't stay at the table	“[he] will get up, get down, stand up, spin around! Needs to be reminded to come back (Carly).”
Concentration issues	“Because of his concentration issues, ever since the kids were little, they eat at separate tables.” (Cath)
Lack of motivation	“Due to time pressure and his own reluctance, we just end up spoon feeding him.” (Cath)
Sensory issues	“This is where we get a lot of gagging. She can't even—the sight the smell, none of it.” (Maddie)
Lengthy mealtimes	“It takes her like over an hour to finish just a standard meal.” (Maddie)
Changing eating patterns	“Every day is a different situation. It feels like it's the lucky wheel of which one are we going to land on today.” (Holly)
Selective eating	“She will eat raw carrots or burnt carrots but not just cooked carrots.” (Holly)
Affected by the environment	“[her brother] is very, very messy. She can't eat near him. If he is in her eye line like she won't eat.” (Cath)
Disengagement with food	“Her almost looking at it for too long. And that's it. She won't eat it.” (Holly)
Need to use certain utensils	“[the grandparents] don't have that cutlery. So then the food's not eaten.” (Cath)
Preference for strong flavours and certain textures	“It either has to be really crunchy, or really salty, or a really sweet fruit.” (Maddie)
Unwilling to try new foods	“Trying to expose her to new food was quite difficult…. she kept discarding meals.” (Alex)
Limited range	“The area that is very, very difficult for me is that he's unwelcoming to eat every food.” (Eric)

### Challenges and strategies

This theme describes how caregivers characterised their child's feeding profile and the practical strategies used to maintain intake, reduce distress, and manage meals in both home and community settings. Caregivers often balanced feeding ideals (shared family meals, dietary variety) against pragmatic constraints (time, resources, child distress). Every caregiver reported their children to have some form of selective eating; they either had a small range of accepted foods, or very specific food preferences. Challenges that differed amongst children were attentional issues, problems with germ contamination, sensory issues, difficulty eating independently, and anxiety around food (see Table 3).

While discussing efforts to encourage eating, every caregiver reported having to prepare special meals for the child; for example, caregivers reported making their children separate meals to what the family was eating, presenting food in a specific manner, or providing a variety of food options. Some strategies had costs and benefits, particularly with the use of initiatives, such as a book or iPad, as Cath noted; “They are often more focused on the book than actually eating. But the advantage is that they’re not distracting each other.” Additionally, some caregivers expressed having to take whatever means necessary to ensure their child consumed something, even if this was not considered ideal. For example, Holly noted, “I let her survive on hot chocolates for 3 days” to ensure that her daughter would consume something when no other preferred food options were available. Such accounts illustrate the degree to some caregivers prioritised short-term intake and emotional wellbeing when faced with periods of intense difficulty, even when this diverged from their nutrition ideals.

Caregivers also took specific measures to address their child's eating in social situations, such as at restaurants or at others' houses. Some caregivers reported feeling limited in their ability to eat out; “I can't remember the last time we all went out and had a meal together” Cath described. One caregiver avoided certain environments, due to the mess and noise. Another reported reduced enjoyment when eating at restaurants, and worried about whether they would offer their child's safe foods; *safe foods* are described by caregivers as food that is typically accepted by the child. Holly reported making alternate food arrangements at other's houses, such as during sleepovers; “I pack her dinner, or I drop her dinner there later to make sure she's eating.” Social participation has often been shaped around their children's *safe foods* and predictable environments.

### Needs for support

This theme encompasses caregivers' experiences navigating service providers, perceived barriers to support, and preference for timely, individualised, and collaborative supports. Caregivers engaged with a wide range of services, including speech therapy, occupational therapy, applied behaviour analysis, dietitians, and school-provided support. Across these services, caregivers frequently differentiated between services that provided validation and practical guidance vs. those perceived as burdensome, inaccessible, or insufficiently responsive.

Experiences with interventions were mixed. Many participants reported improvement in some areas of their child's feeding difficulties, but also discussed behaviours that were persistent and unchanged, such as length of mealtime and openness to new foods. For example, Eric said, “We’re seeing a therapist every week… but yet still I don't see any changes.” Factors that limited access to services included distance, expense, small program intake, and time. There were also perceived barriers; for a few caregivers, the stress associated with seeking out or undergoing services is a barrier for families. One caregiver delayed diagnosis seeking, noting that pathology procedures could be particularly traumatising for her child. Holly also exhibited uncertainty about the value of having a diagnosis: “So then, is it ARFID [Avoidant/Restrictive Food Intake Disorder], or is it something else?… I’m not very big on the labels unless it's going to help.” For these caregivers, diagnostic labels were evaluated instrumentally—valuing them only insofar as they improved access to services or clarified actionable pathways. For some caregivers, a perceived barrier was not being able to talk about their child's feeding difficulties due to judgement from others. Maddie reported not knowing how to describe her child's issues accurately, due to their variability: “When I complete a questionnaire around ‘what does your kid eat’, It's hard, because she kind of eats everything, but nothing.” This speaks to the diverse and changing challenges these caregivers have with their child's feeding that translate to challenges in how to access the right service.

Every caregiver expressed highly specific needs for support regarding their child's feeding, and many suggested specific tools and strategies that they would find helpful. Examples included the provision of personalised meal ideas and routine schedules for eating, nutrition monitoring, timely feedback from therapists, and a shared platform with other caregivers with similar issues. Overall, an emphasis was placed on wanting collaborative help, either with a therapist or other caregivers. For example, Maddie experienced difficulties with implementing techniques at home without being able to consult their therapist between sessions: “It confuses her [child], and I then don't have a lot of credibility to follow through on anything thereafter.” Some caregivers reported the value of hearing from other caregivers with similar experiences, who could share difficulties and strategies. Additionally, both fathers explicitly sought ways to better their relationship with their child and valued interventions that would address their own frustration and anxiety around mealtimes arising from difficult eating behaviours.

Caregivers also had conflicting views on the provision of telehealth services as a way of meeting these gaps. Several noted that they would be convenient, particularly when travel was an issue: Eric said, “I can be in my comfort zone and not have to pass through the stress of transportation.” A few caregivers suggested that telehealth would be helpful as an observational tool, for example, to naturalistically examine the child's eating and behaviour. However, most agreed that it would not be feasible for services to be delivered directly to the child virtually; caregivers reported concerns around the therapist's ability to get to know the child and conduct activities online based on past experiences. There were also issues with children's engagement with telehealth: “If she [child] doesn't want to participate, which a lot of the time she doesn't, she will just walk away”, Holly described. Overall, caregivers framed telehealth as potentially useful for reducing access barriers and supporting observation, but less acceptable for direct child-facing intervention when engagement and rapport were concerns.

## Discussion

This exploratory qualitative study examined how caregivers describe the impacts of feeding difficulties on family life and their perceived support needs. Four themes emerged from the framework analysis: parental concern for child wellbeing, impacts on the family, challenges and strategies used to manage feeding, and needs for support. Together, these findings illustrate that feeding difficulties are experienced by caregivers not only as nutritional concerns, but as complex relational and emotional challenges embedded within family life.

### Impacts on the child and family

The impacts of the feeding difficulty on the child's nutrition were a primary concern for caregivers in the current study, consistent with findings from previous research (8, 12). Consistent with previous research, selective eating was the most reported feeding challenge amongst caregivers (11, 12). Caregivers in the present study, however, framed this as nutritional challenges and importantly highlighted these issues affect social participation and wellbeing. A key contribution of this study is the identification of caregivers' concerns about the broader psychosocial impact of their child's feeding such as in school, or impact their relationships with others. Similarly to Simione et al. (18), some caregivers reported that their child required extra support during feeding, such as being fed or prompted to eat. While children in the study by Simione et al. were aged two to five, findings of the present study indicate that supervision and physical help during feeding is sometimes required for older children too. Caregivers also discussed their children's awareness of their own feeding difficulties, and worried about how they process these issues. This finding highlights the potential emotional impacts of having feeding difficulties and emphasises the need to gain the child's perspective. Limited studies have directly involved the child in examination of these issues, as these issues may be difficult to articulate. Previously, research has addressed this by examining the experiences of older children; Wolstenholme et al. (19) investigated how children 6–10 years old perceived, experienced and managed feeding difficulties. Findings indicated that children were highly perceptive of their caregiver's strategies to encourage eating and struggled to manage their internal experiences—or reasons for being fussy, with external expectations of their eating (19). Future research should seek to gain a more nuanced understanding of the child perspective on this issue across a broader age range and with neurodiverse populations.

Caregivers also spoke considerably about the impacts of their child's eating habits on their own well-being and that of the broader family. Caregivers report that mealtimes induced stress, anxiety, and frustration around certain fussy behaviours, such as food waste—in line with previous research (8, 10, 11). Managing mealtimes and pursuing interventions was particularly overwhelming for caregivers of multiple children with differing feeding needs, some of whom are full-time caregivers. Some children were reported to exhibit feeding difficulties behaviour that was disruptive to mealtimes, or emitted strategies that were disruptive. For example, strategies such as making alternate meal arrangements or eating separately from their children were commonly reported by caregivers in this study and in that by Trofholz et al. (11). These findings reinforce child feeding difficulties as a family systems issue rather than solely a child behaviour challenge. Mealtime management requires considerable emotional and logistical labour from caregivers, often impacting sibling and parent wellbeing.

Another impact on the family was that caregivers had reduced time to spend with siblings without feeding difficulties. Schumann et al. (20) reported that siblings of a child with a neurodevelopmental disorder felt reduced emotional and physical availability from their caregivers, and restriction when it came to family activities such as going out for a meal. While the present study investigated this issue from the perspective of caregivers rather than siblings, findings provide insight into the secondary effects of child feeding difficulties on others in the family. Three of the eight children in this study reported a diagnosed neurodevelopmental disorder, and caregivers noted negative impacts of feeding difficulties on siblings.

Notably, caregivers reported some generational difference in attitudes towards feeding. All caregivers showed understanding of their child's difficulties, and some discussed having a more flexible mindset towards mealtime rules than their own parents. These dynamics may relate to broader cultural differences in feeding practices (21, 22); however, the present study did not directly examine cultural background meaning these interpretations should be considered tentative. The present study did find that caregivers valued their own feeding practices and their child's preferences more than adapting to meet the expectations of grandparent. Future research should explicitly explore generational and cultural implications of feeding.

### Implications for support services

This study provides insight into caregiver's attitudes, modality of feeding and strategies used to address child difficulties. Caregivers valued and encouraged their child's autonomy; however, they also expressed a need to get their child fed by any means and sometimes used methods they considered unfavourable out of necessity. For a few caregivers, this involved coercive strategies—such as physically feeding children to speed up eating or using incentives, and many caregivers used practices such as making the child special meals or offering any food they will accept, similar to reports by Chilman et al. (10) and Harris et al. (5). Coercive strategies may interfere with a child's ability to self-regulate food intake, while indulgent feeding can result in lower consumption of nutritionally appropriate foods and an increased chance of becoming overweight (1, 9). Interventions should teach child-centred and responsive feeding practices (e.g., 1); however, practically this can be difficult for caregivers of children who refuse to or lack the motivation to eat. Therefore, feeding interventions should align evidence-based practices with the family context given the complexity of feeding in the home.

Caregivers requested a need for support across a range of feeding domains. Previous studies conducted in Spain and Sweden indicate that many caregivers felt that their needs were undervalued by healthcare professionals (6, 7). These studies sampled caregivers of children with diagnosed feeding disorders and encompassed their experiences of paediatric and hospital care; whereas the present study explored feeding difficulties more broadly, and caregivers discussed their experiences with a range of services. While we did not ask caregivers in the present study if they felt dismissed by service providers, levels of satisfaction with healthcare professionals were mixed. Caregivers reported feeling that their expectations were not met in terms of improvement of feeding difficulties in their child, and some did not feel adequately supported in carrying out interventions independently. Caregivers sought clear and simplified strategies and increased communication from health professionals. Additionally, caregivers expressed the desire for collaboration with other caregivers—feedback from others was considered helpful in recognising feeding difficulties in their own children and sharing tips to manage difficulties. This is consistent with Fraser et al. (12), who conducted a content analysis of caregiver help-seeking behaviour on an online forum. Accordingly, therapist-facilitated support groups may be useful for allowing caregivers to connect with others, and providing professional guidance.

In terms of barriers, travel time, expense, small intake, and the stress associated with seeking these services were reported to be issues. Although telehealth has been proposed as a way to address these barriers (23, 24), caregivers in the present study reported mixed experiences with receiving feeding support online, which caused apprehension about engagement with telehealth. While remote service delivery was reported to be convenient for caregivers, caregivers still questioned its effectiveness in engaging their children in intervention. Another barrier was difficulty in describing behaviours relating to feeding difficulties; as caregiver reports are commonly used to measure feeding difficulties (25); thus, interviews may be more comprehensive at understanding the family need than many existing questionnaires for clinical intake.

### Limitations

The sample of this study was small, heterogeneous, yet limited in diversity; this is a caveat to consider regarding the generalisability of findings. The broad age range of children in this study allowed for diverse caregiver experiences; however, does not allow for the study of developmental differences in feeding difficulties. Recruitment was partially undertaken through Australian support providers, including disability clinics, which may relate to the number of children with a psychological diagnosis in this study (50%). This has implications for the study's representativeness of the general population of children who exhibit feeding difficulties, although the prevalence of atypical eating behaviour is significantly higher in children with developmental disabilities than in neurotypical children (26). Additionally, some socio-demographic data—such as age and ethnicity, was not reported, which impacted the ability to contextualise the experiences of participants. Australia is immensely diverse—more than half of Australian residents are culturally and linguistically diverse, and 3.2% of the population consists of First Nations people (27), however, the diversity of this sample could not be discussed as data on ethnicity was not collected. Future studies should seek to gain a more comprehensive understanding of how feeding practice may vary across different cultural groups in Australia.

While this study investigated the experiences of both mothers (*n* = 4) and fathers (*n* = 2), no interviews were undertaken with parent dyads nor caregivers of other primary caregivers outside the *mother* or *father* role. Examining the perspective of the co-parenting dyad may provide a more nuanced understanding of their experiences, especially regarding potential differences in feeding techniques. Further, the perspectives of caregivers other than mothers and fathers were not explored in this sample.

## Conclusion

Overall, the findings suggest that feeding difficulties are experienced by caregivers as complex relational challenges that extend beyond nutrition into family functioning, emotional wellbeing, and service navigation. The present study contributes to the understanding of caregiver experiences of raising a child with feeding difficulties. Findings speak to the nutritional and psychosocial impacts of feeding difficulties on the child as well as the consequences of the child's feeding difficulties on the family. Interventions should therefore take a family-centred approach to support caregivers address feeding difficulties and reduce mealtime stress. Caregivers also report a need for practical and actionable support from services, emphasising the importance of collaborative care and transferrable skills in the home setting. Considering the barriers to obtaining services, telehealth may offer a promising avenue for caregiver-training, but future research should investigate the child perspective of these services.

## Data Availability

The raw data supporting the conclusions of this article will be made available by the authors, without undue reservation.
